# Clinical outcomes of first-line nivolumab plus chemotherapy in patients aged ≥ 75 years with unresectable advanced or recurrent gastric cancer: a retrospective study

**DOI:** 10.1186/s12957-026-04525-x

**Published:** 2026-08-03

**Authors:** Masaki Yoshimatsu, Shinji Morita, Yu Ueta, Minori Mise, Noboru Inoue, Takahiro Ochiai, Shuhei Takise, Junki Fujita, Hiroto Muroi, Masatoshi Nakagawa, Masanobu Nakajima, Satoru Yamaguchi, Kazuyuki Kojima

**Affiliations:** 1 Department of Upper Gastrointestinal Surgery, Dokkyo Medical University, 880 Kitakobayashi, Mibu, Tochigi, 321-0293 Japan; 2 Department of Surgery, Dokkyo Medical University Nikko Medical Center, Tochigi, Japan

**Keywords:** Stomach neoplasms, Nivolumab, Immune checkpoint inhibitors, Aged, Retrospective studies

## Abstract

**Background:**

Older patients with unresectable advanced or recurrent gastric cancer remain underrepresented in clinical trials of immune checkpoint inhibitors. This study assessed the clinical feasibility of first-line nivolumab plus chemotherapy in patients aged ≥ 75 years treated at our institutions.

**Methods:**

This retrospective study included 82 patients with HER2-negative unresectable advanced or recurrent gastric cancer who received first-line nivolumab-containing chemotherapy between January 2022 and February 2025. Patients were divided into two groups: those aged < 75 years (*n* = 52) and those aged ≥ 75 years (*n* = 30). The primary endpoint was progression-free survival (PFS); secondary endpoints included overall survival (OS), objective response rate (ORR), disease control rate (DCR), and adverse events (AEs).

**Results:**

Patients aged ≥ 75 years more frequently had comorbidities, hypertension, and initial dose reductions. Median follow-up was 27.7 months in the < 75 years group and 24.3 months in the ≥ 75 years group. Median PFS was 7.2 months in the < 75 years group and 5.6 months in the ≥ 75 years group (*P* = 0.779). Median OS was 12.5 and 13.3 months, respectively (*P* = 0.852). No significant differences were observed in ORR, DCR, or AEs between the two groups.

**Conclusions:**

These findings suggest that first-line nivolumab plus chemotherapy may represent a clinically feasible treatment option for selected patients aged ≥ 75 years with HER2-negative, unresectable, advanced or recurrent gastric cancer. Nevertheless, given the limited sample size, these results should be interpreted with caution and should not be construed as evidence of equivalent efficacy or safety between age groups.

## Background

In Japan, patients aged ≥ 75 years accounted for 58% of newly diagnosed gastric cancer cases in 2023 and 72% of gastric cancer-related deaths in 2024 [1]. Older patients often present with multiple comorbidities and age-related functional decline, complicating treatment selection [2, 3]. Optimizing therapeutic strategies for this population has therefore become an increasingly important issue in contemporary clinical practice.

Significant advances have recently been made in the systemic treatment of unresectable advanced or recurrent gastric cancer, and immune checkpoint inhibitor (ICI)-based combination chemotherapy has emerged as a standard first-line option [4–6]. However, age-associated alterations in immune function—collectively referred to as immunosenescence—have been hypothesized to attenuate the efficacy of ICIs in older patients [3, 7]. Pivotal trials evaluating ICI-containing first-line regimens enrolled few older patients, and age-based subgroup analyses were not powered to draw definitive conclusions for this population. Notably, patients aged ≥ 75 years constituted only approximately 6% of the CheckMate 649 population [3, 5, 6]. Consequently, evidence specifically addressing the efficacy and safety of ICI-containing first-line therapy in patients aged ≥ 75 years remains limited.

Current treatment guidelines and expert consensus statements emphasize individualized therapeutic strategies informed by age-related factors. Rather than relying solely on chronological age, systemic therapy in older patients should be selected through comprehensive assessment of comorbidities, functional status, and physiological reserve [2, 3]. In Japan, individuals aged ≥ 75 years are categorized as a distinct subgroup in geriatric medicine and health policy, reflecting their specific clinical characteristics and healthcare needs; the Joint Committee of the Japan Gerontological Society and the Japan Geriatrics Society has formally proposed redefining “elderly” as those aged ≥ 75 years [8].

Against this background, we evaluated the clinical feasibility and outcomes of nivolumab-containing first-line chemotherapy in older patients (aged ≥ 75 years) compared with younger patients with unresectable advanced or recurrent gastric cancer with the aim of clarifying whether advanced age alone should preclude the use of this regimen in routine clinical practice. To our knowledge, studies specifically evaluating ICI-containing first-line chemotherapy in patients aged ≥ 75 years with gastric cancer in real-world settings remain limited.

## Methods

### Study design and patients

**Fig. 1:**
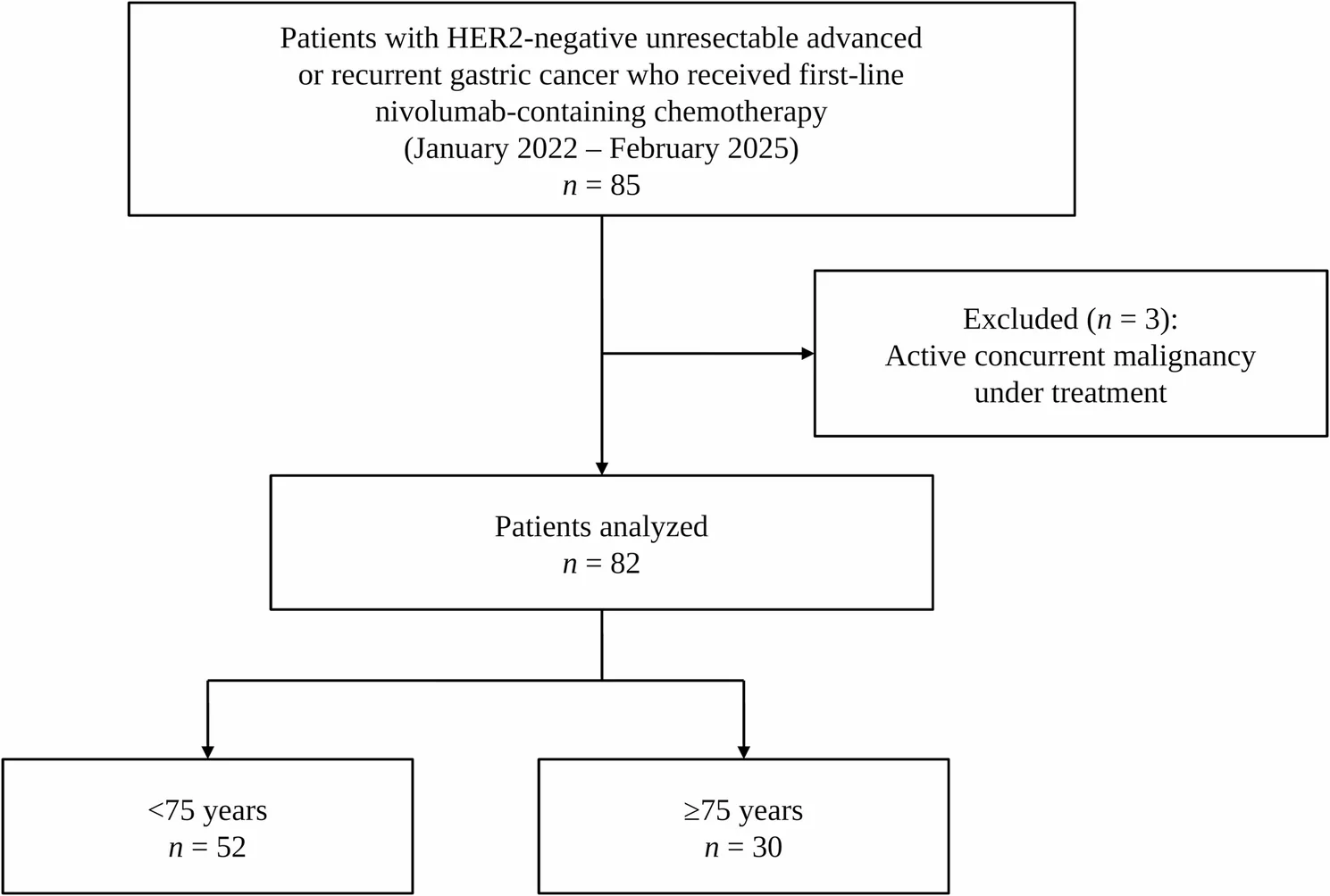
Flow diagram of patient selection. Of 85 patients with HER2-negative unresectable advanced or recurrent gastric cancer who received first-line nivolumab-containing chemotherapy between January 2022 and February 2025, three were excluded because of an active concurrent malignancy under treatment, leaving 82 patients for analysis (52 aged < 75 years and 30 aged ≥ 75 years)

This retrospective study evaluated the safety and efficacy of first-line nivolumab combined with fluoropyrimidine- and platinum-based chemotherapy in patients with HER2-negative unresectable advanced or recurrent gastric cancer treated at Dokkyo Medical University Hospital and Dokkyo Medical University Nikko Medical Center between January 2022 and February 2025 (Fig. 1).

Eligible patients had histologically confirmed disease and received nivolumab-containing first-line chemotherapy. All patients were HER2-negative and HER2 status was assessed in all patients as part of routine first-line treatment selection. Patients who had received preoperative chemoradiotherapy were excluded. Prior or concurrent malignancy was not an exclusion criterion when clinically controlled and not expected to interfere with the evaluation of treatment efficacy or safety. Accordingly, one patient with a history of surgically treated, well-controlled prostate cancer was included. Patients were categorized into two groups: those aged < 75 years and those aged ≥ 75 years. At our institutions, nivolumab-containing combination chemotherapy was considered for patients in whom Eastern Cooperative Oncology Group (ECOG) performance status (PS), organ function, comorbidities, oral intake, and patient preference were deemed compatible with combination therapy. For older or potentially vulnerable patients, upfront dose reduction was routinely employed to facilitate treatment initiation, rather than excluding such patients from combination therapy on the basis of chronological age alone.

Clinical data, including patient demographics, ECOG PS, tumor location, histological subtype, programmed death-ligand 1 (PD-L1) combined positive score (CPS), comorbidities, disease status, sites of metastasis, treatment regimens, and clinical outcomes, were retrospectively collected from medical records. Histological type was classified according to the Lauren classification. Baseline laboratory data and inflammatory/nutritional indices were also assessed, including the neutrophil-to-lymphocyte ratio (NLR), platelet-to-lymphocyte ratio (PLR), prognostic nutritional index (PNI), modified Glasgow prognostic score (mGPS), and geriatric nutritional risk index (GNRI).

The study protocol was approved by the Institutional Review Board of Dokkyo Medical University (approval number: R-105–4 J). This study was conducted in accordance with the Declaration of Helsinki. The requirement for informed consent was waived owing to the retrospective nature of the study, and information about the study was disclosed using an opt-out approach.

### Treatment regimens

Patients received nivolumab in combination with one of the following regimens: 

#### SOX plus nivolumab

Oxaliplatin (100 mg/m²) and nivolumab (360 mg) were administered intravenously on day 1, and S-1 (80 mg/m²/day) was administered orally on days 1–14 of a 21-day cycle. 

#### CapeOX plus nivolumab

Oxaliplatin (130 mg/m²) and nivolumab (360 mg) were administered intravenously on day 1, and capecitabine (2000 mg/m²/day) was administered orally on days 1–14 of a 21-day cycle. 

#### FOLFOX plus nivolumab

Nivolumab (240 mg) was administered intravenously together with oxaliplatin (85 mg/m²) and leucovorin (200 mg/m²), followed by a bolus of 5-fluorouracil (400 mg/m²) and a 46-hour continuous infusion of 5-fluorouracil (2400 mg/m²), repeated every 14 days.

Treatment was continued until disease progression, unacceptable toxicity, clinical deterioration, or withdrawal of consent.

### Endpoints and assessments

The primary endpoint was progression-free survival (PFS), defined as the time from treatment initiation to disease progression or death from any cause. Secondary endpoints included overall survival (OS), objective response rate (ORR), disease control rate (DCR), and adverse events (AEs), including immune-related AEs (irAEs).

Tumor response was assessed by computed tomography at baseline and at regular intervals until treatment discontinuation, in accordance with Response Evaluation Criteria in Solid Tumors (RECIST) version 1.1. Adverse events were graded according to the Common Terminology Criteria for Adverse Events (CTCAE) version 5.0.

### Statistical analysis

Continuous variables were compared using the Mann–Whitney U test, and categorical variables were compared using the chi-square or Fisher’s exact test, as appropriate. Survival outcomes were estimated using the Kaplan–Meier method and compared using the log-rank test. In the ≥ 75 years subgroup, exploratory univariable and multivariable Cox proportional hazards analyses were performed. Multivariable models were limited to two covariates given the limited number of events in the ≥ 75 years subgroup. Variables were selected based on clinical relevance and univariable analysis results. The proportional hazards assumption in the Cox models was assessed graphically using log-minus-log survival plots. Hazard ratios (HRs) and 95% confidence intervals (CIs) were calculated. All tests were two-sided, and *P* < 0.05 was considered statistically significant. All analyses were performed using SPSS version 30.0 (IBM Corp., Armonk, NY, USA). No formal sample size calculation was performed, as this study was retrospective and exploratory in nature. The study was not powered to formally test equivalence between age groups, and all between-group comparisons should be interpreted as hypothesis-generating.

## Results

### Baseline characteristics

Baseline characteristics are summarized in Table 1. Among the 82 patients included, 52 were aged < 75 years and 30 were aged ≥ 75 years, with median ages of 69 years (range, 41–74) and 79 years (range, 75–86), respectively. The sex distribution differed significantly between groups, with a higher proportion of male patients in the ≥ 75 years group (90.0% vs. 65.4%; *P* = 0.014). Height, body weight, and body mass index were comparable between groups. ECOG performance status of 2–4 was numerically more frequent in patients aged ≥ 75 years (13.3% vs. 1.9%; *P* = 0.057). Lauren classification and PD-L1 CPS did not differ significantly between groups. CPS was unavailable in some patients because tumor specimens had been obtained at external institutions or because CPS testing had not been performed at the time of diagnosis. Disease status (recurrent vs. unresectable) was also comparable (*P* = 0.289). Regarding comorbidities, any comorbidity was more prevalent in the ≥ 75 years group (90.0% vs. 71.2%; *P* = 0.047), with hypertension being significantly more common (63.3% vs. 36.5%; *P* = 0.019). Peritoneal metastasis was less frequent in patients aged ≥ 75 years (43.3% vs. 67.3%; *P* = 0.034).

**Table 1:** Baseline characteristics

Factors	< 75 years (*n* = 52)	≥ 75 years (*n* = 30)	*P*-value
Demographics and clinical status			
Age, years	69 (41–74)	79 (75–86)	–
Sex, male: female	34:18	27:3	0.014
Height, m	1.62 (1.45–1.78)	1.62 (1.42–1.79)	0.682
Body weight, kg	56.7 (34.6–85.8)	55.7 (40.4–73.0)	0.651
BMI, kg/m²	21.5 (14.4–35.1)	21.2 (16.8–26.9)	0.954
ECOG PS, 0–1:2–4	51:1	26:4	0.057
Tumor location, U:M: L	17:18:17	7:10:13	0.558
Lauren classification, Intestinal: Diffuse: Unknown	17:32:3	16:14:0	0.103
PD-L1 expression CPS, < 5:≥5:unknown	26:19:7	12:13:5	0.431
Disease status, Recurrent: Unresectable	14:38	5:25	0.289
Comorbidities			
Any comorbidity	37 (71.2%)	27 (90.0%)	0.047
Cardiovascular disease	10 (19.2%)	9 (30.0%)	0.266
Cerebrovascular disease	3 (5.8%)	3 (10.0%)	0.664
Diabetes mellitus	10 (19.2%)	6 (20.0%)	0.933
Hypertension	19 (36.5%)	19 (63.3%)	0.019
Renal dysfunction	1 (1.9%)	3 (10.0%)	0.136
Respiratory disease	4 (7.7%)	0 (0%)	0.291
Patterns of metastasis			
Hematogenous metastasis	20 (38.4%)	11 (36.7%)	0.872
Distant lymph node metastasis	11 (21.2%)	9 (30.0%)	0.369
Peritoneal metastasis	35 (67.3%)	13 (43.3%)	0.034

Values are presented as median (range), *n* (%), or n unless otherwise indicated

Hematogenous metastasis included liver, bone, lung, and brain metastases. Categories were not mutually exclusive

*BMI *body mass index, *ECOG PS* Eastern Cooperative Oncology Group performance status, *PD-L1 CPS *programmed death-ligand 1 combined positive score

### Laboratory data

Among laboratory data (Table 2), serum cholinesterase levels were significantly lower (172 vs. 203 U/L; *P* = 0.032) and PLR levels were also significantly lower (173.0 vs. 223.1; *P* = 0.047) in the ≥ 75 years group. Albumin, total protein, NLR, PNI, mGPS, and GNRI were comparable between groups.

**Table 2:** Laboratory data

Factors	< 75 years (*n* = 52)	≥ 75 years (*n* = 30)	*P*-value
Alb, g/dL	3.5 (1.7–4.6)	3.3 (1.9–4.3)	0.076
ChE, U/L	203 (85–436)	172 (92–394)	0.032
TP, g/dL	6.6 (4.7–8.0)	6.5 (3.9–7.6)	0.405
NLR	3.26 (1.22–27.10)	3.27 (0.94–9.44)	0.303
PLR	223.1 (43.0–893.5)	173.0 (63.4–543.6)	0.047
PNI	40.3 (19.6–59.6)	40.6 (23.4–49.1)	0.129
mGPS, 0:1:2	30:6:16	18:1:11	0.423
GNRI category, No: Low: Moderate: Major	13:5:17:17	3:4:11:12	0.422

Values are presented as median (range), *n* (%), or n unless otherwise indicated

*Alb* albumin, *ChE* cholinesterase, *TP* total protein, *NLR* neutrophil-to-lymphocyte ratio, *PLR* platelet-to-lymphocyte ratio, *PNI* prognostic nutritional index, *mGPS* modified Glasgow prognostic score, *GNRI * geriatric nutritional risk index

### Treatment exposure

Treatment exposure is summarized in Table 3. The median number of nivolumab combination cycles was similar between groups (7 [range, 1–32] vs. 6 [range, 1–28]; *P* = 0.445), and the distribution of chemotherapy backbones (SOX, CapeOX, or FOLFOX) did not differ significantly (*P* = 0.955). However, initial dose reductions of fluoropyrimidines and oxaliplatin were significantly more frequent in the ≥ 75 years group than in the < 75 years group (76.7% vs. 36.5%, *P* < 0.001 and 60.0% vs. 30.8%, *P* = 0.010, respectively). The relative dose intensity per cycle—defined as the administered dose as a proportion of the planned dose, averaged across all administered cycles and excluding dose delays or treatment interval extensions—was significantly lower in the ≥ 75 years group for both fluoropyrimidines (median, 78.8% vs. 89.3%; *P* = 0.002) and oxaliplatin (median, 77.1% vs. 89.3%; *P* = 0.004). Reasons for discontinuation of first-line treatment are summarized in Table 3. Disease progression was the most common reason in both groups (44.2% vs. 43.3%). Treatment-related toxicity accounted for treatment discontinuation in 28.8% and 20.0% of patients in the < 75 years and ≥ 75 years groups, respectively, and the overall distribution of discontinuation reasons did not differ significantly between groups (*P* = 0.682). Conversion surgery was performed in four patients (two in each group). Second-line therapy consisted predominantly of paclitaxel plus ramucirumab in both groups (48.1% vs. 40.0%), with no significant between-group difference in the distribution of subsequent therapies (*P* = 0.280).

**Table 3:** Treatment exposure

Factors	< 75 years (*n* = 52)	≥ 75 years (*n* = 30)	*P*-value
Median nivolumab combination cycles	7 (1–32)	6 (1–28)	0.445
Combination regimen, SOX: CapeOX: FOLFOX	40:6:6	23:3:4	0.955
Initial fluoropyrimidine dose reduction	19 (36.5%)	23 (76.7%)	< 0.001
Relative dose intensity per cycle, fluoropyrimidine	89.3 (80.0–99.3)	78.8 (67.0–83.0)	0.002
Initial oxaliplatin dose reduction	16 (30.8%)	18 (60.0%)	0.010
Relative dose intensity per cycle, oxaliplatin	89.3 (80.0–100)	77.1 (60.0–80.7)	0.004
Reason for first-line discontinuation			0.682
Ongoing without progression	4 (7.7%)	4 (13.3%)	
Disease progression	23 (44.2%)	13 (43.3%)	
Toxicity	15 (28.8%)	6 (20.0%)	
Declining performance status	3 (5.8%)	4 (13.3%)	
Patient preference	4 (7.7%)	1 (3.3%)	
Death	1 (1.9%)	0 (0%)	
Conversion surgery	2 (3.8%)	2 (6.7%)	
Subsequent (second-line) therapy			0.280
Ongoing without progression	7 (13.5%)	7 (23.3%)	
Paclitaxel plus ramucirumab	25 (48.1%)	12 (40.0%)	
Other	7 (13.5%)	1 (3.3%)	
None (best supportive care or death)	13 (25.0%)	10 (33.3%)	

Values are presented as median (range), *n* (%), or n

*SOX* S-1 plus oxaliplatin, *CapeOX* capecitabine plus oxaliplatin, *FOLFOX* leucovorin, fluorouracil, and oxaliplatin

### Safety

Safety outcomes are summarized in Table 4. No statistically significant between-group differences were observed in the incidence of any AE (84.6% vs. 80.0%; *P* = 0.593), grade ≥ 3 AE (40.4% vs. 53.3%; *P* = 0.256), any irAE (30.8% vs. 20.0%; *P* = 0.289), or grade ≥ 3 irAE (17.3% vs. 6.7%; *P* = 0.195). Similarly, no significant differences were observed in the incidence of grade ≥ 3 hematologic AEs (19.2% vs. 33.3%; *P* = 0.152) or grade ≥ 3 nonhematologic AEs (28.8% vs. 23.3%; *P* = 0.587). Among individual grade ≥ 3 AEs, no nonhematologic event reached statistical significance, whereas grade ≥ 3 thrombocytopenia was numerically more frequent in the ≥ 75 years group (13.3% vs. 1.9%; *P* = 0.057). Among grade ≥ 3 irAEs, interstitial pneumonitis occurred only in the < 75 years group (7.7% vs. 0%; *P* = 0.291), though this difference was not statistically significant.

**Table 4:** Treatment-related adverse events

	< 75 years (*n* = 52)	≥ 75 years (*n* = 30)	*P*-value
Any AE	44 (84.6%)	24 (80.0%)	0.593
Grade ≥ 3 AE	21 (40.4%)	16 (53.3%)	0.256
Grade ≥ 3 nonhematologic AE	15 (28.8%)	7 (23.3%)	0.587
Grade ≥ 3 hematologic AE	10 (19.2%)	10 (33.3%)	0.152
Any irAE	16 (30.8%)	6 (20.0%)	0.289
Grade ≥ 3 irAE	9 (17.3%)	2 (6.7%)	0.195
Selected grade ≥ 3 hematologic AEs			
Neutropenia	5 (9.6%)	4 (13.3%)	0.718
Leukopenia	2 (3.8%)	1 (3.3%)	1.000
Anemia	5 (9.6%)	2 (6.7%)	1.000
Thrombocytopenia	1 (1.9%)	4 (13.3%)	0.057
Selected grade ≥ 3 irAEs			
Interstitial pneumonitis	4 (7.7%)	0 (0%)	0.291
Adrenal insufficiency	2 (3.8%)	1 (3.3%)	1.000
Enterocolitis	1 (1.9%)	1 (3.3%)	1.000
Hepatitis	2 (3.8%)	0 (0%)	0.530

Values are presented as *n* (%)

*CTCAE* Common Terminology Criteria for Adverse Events, *AE* adverse event, *irAE* immune-related adverse event

### Tumor response and survival outcomes

**Fig. 2:**
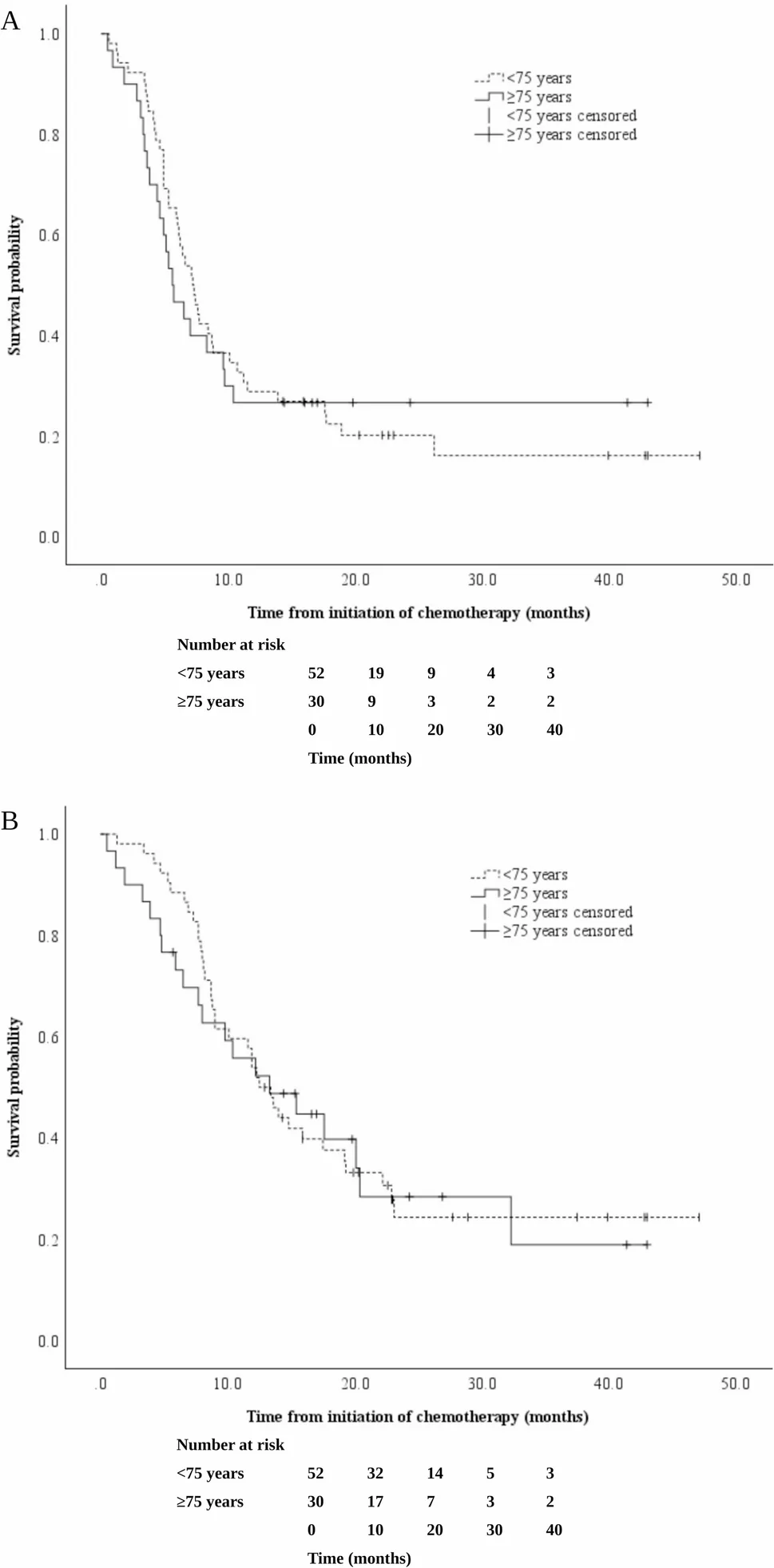
Kaplan–Meier curves for survival outcomes according to age group. **A** Progression-free survival. **B** Overall survival. Tick marks indicate censored observations. Numbers at risk are shown below each panel

Tumor response and survival outcomes are summarized in Table 5. Median follow-up, estimated by the reverse Kaplan–Meier method, was 27.7 months (95% CI, 18.5–36.9) in the < 75 years group and 24.3 months (95% CI, 15.4–33.2) in the ≥ 75 years group. Events occurred in 42 of 52 patients (< 75 years) and 22 of 30 patients (≥ 75 years) for PFS, and in 37 of 52 and 20 of 30 for OS. Best overall response did not differ significantly between groups (*P* = 0.452). ORR was 40.4% in the < 75 years group and 46.7% in the ≥ 75 years group (*P* = 0.580); DCR was 92.3% and 86.7%, respectively (*P* = 0.455). Among patients with known CPS, ORR in the ≥ 75 years group was 41.7% in CPS < 5 and 61.5% in CPS ≥ 5 (*P* = 0.320). Given the substantial proportion of patients with unknown CPS in the ≥ 75 years group (16.7%), these analyses should be interpreted with caution. Median PFS was 7.2 months (95% CI, 5.8–8.6) in the < 75 years group and 5.6 months (95% CI, 3.7–7.5) in the ≥ 75 years group (*P* = 0.779) (Fig. 2A). Median OS was 12.5 months (95% CI, 10.1–14.9) and 13.3 months (95% CI, 5.1–21.5), respectively (*P* = 0.852) (Fig. 2B).

**Table 5:** Tumor response and survival outcomes

	< 75 years (*n* = 52)	≥ 75 years (*n* = 30)	*P*-value
Best overall response, CR: PR: SD: PD	4:17:27:4	5:9:12:4	0.452
ORR	21 (40.4%)	14 (46.7%)	0.580
DCR	48 (92.3%)	26 (86.7%)	0.455
Median PFS, months (95% CI)	7.2 (5.8–8.6)	5.6 (3.7–7.5)	0.779
Median OS, months (95% CI)	12.5 (10.1–14.9)	13.3 (5.1–21.5)	0.852

Values are presented as *n* (%) or median (95% confidence interval)

*CI *confidence interval, *CR* complete response, *PR* partial response, *SD* stable disease, *PD* progressive disease, *ORR* objective response rate, *DCR* disease control rate, *PFS* progression-free survival, *OS* overall survival

Exploratory Cox analyses were performed within the ≥ 75 years subgroup (Table 6). In univariable analyses for PFS, higher NLR was associated with shorter PFS (HR, 1.524; 95% CI, 1.203–1.930; *P* < 0.001), and higher total protein was associated with longer PFS (HR, 0.617; 95% CI, 0.399–0.953; *P* = 0.030). In multivariable analysis, NLR remained significantly associated with shorter PFS after adjustment for total protein (HR, 1.454; 95% CI, 1.134–1.866; *P* = 0.003), whereas total protein was no longer significant (HR, 0.741; 95% CI, 0.462–1.186; *P* = 0.211). For OS, univariable analyses identified diffuse-type Lauren classification (HR, 4.155; 95% CI, 1.552–11.122; *P* = 0.005) and higher total protein (HR, 0.575; 95% CI, 0.347–0.952; *P* = 0.032) as significant factors. In multivariable analysis, diffuse-type Lauren classification remained significantly associated with shorter OS after adjustment for total protein (HR, 3.451; 95% CI, 1.206–9.881; *P* = 0.021), whereas total protein was no longer significant (HR, 0.742; 95% CI, 0.444–1.239; *P* = 0.254). Log-minus-log survival plots showed no substantial violation of the proportional hazards assumption for any covariate included in the multivariable models.

**Table 6:** Exploratory univariable and multivariable cox analyses in the ≥ 75 years subgroup (*n* = 30)

	Univariable analysis			Multivariable analysis		
	HR	95% CI	*P*-value	HR	95% CI	*P*-value
Progression-free survival						
NLR	1.524	1.203–1.930	< 0.001	1.454	1.134–1.866	0.003
Total protein	0.617	0.399–0.953	0.030	0.741	0.462–1.186	0.211
Lauren classification (Diffuse vs. Intestinal)	2.354	0.999–5.548	0.050	–	–	–
Overall survival						
NLR	1.145	0.954–1.375	0.145	–	–	–
Total protein	0.575	0.347–0.952	0.032	0.742	0.444–1.239	0.254
Lauren classification (Diffuse vs. Intestinal)	4.155	1.552–11.122	0.005	3.451	1.206–9.881	0.021

Values are presented as hazard ratio (HR), 95% confidence interval (CI), or *P*-value unless otherwise indicated

*NLR* neutrophil-to-lymphocyte ratio, *OS* overall survival, *PFS* progression-free survival

## Discussion

The principal finding of this study was that no statistically significant differences were observed in PFS, OS, tumor response, or safety outcomes between older patients (aged ≥ 75 years) and younger patients (aged < 75 years), despite a significantly higher prevalence of comorbidities and more frequent initial dose reductions in the older cohort. Given the limited sample size of the ≥ 75 years cohort and the resulting wide confidence intervals, these comparisons should be regarded as hypothesis-generating.

The efficacy outcomes observed in this study were broadly consistent with those reported in previous real-world studies of ICI-containing first-line chemotherapy for gastric cancer [9]. The ORR was 40.4% in the < 75 years group and 46.7% in the ≥ 75 years group (*P* = 0.580), and the DCR was 92.3% and 86.7%, respectively (*P* = 0.455). Although these results were numerically lower than those reported in pivotal phase 3 trials such as CheckMate 649 and ATTRACTION-4 [5, 6], direct cross-study comparisons should be interpreted with caution. The overall pattern was nonetheless consistent with prior real-world data, including the Japanese multicenter G-KNIGHT study [10]. Notably, patients aged ≥ 75 years comprised only approximately 6% of the CheckMate 649 population, and dedicated efficacy data for this age group remain scarce; our findings extend the available real-world evidence to this underrepresented population and are broadly concordant with recent real-world analyses that included older patients [9, 10]. Median PFS was 7.2 months in the < 75 years group and 5.6 months in the ≥ 75 years group (*P* = 0.779), and median OS was 12.5 months and 13.3 months, respectively (*P* = 0.852). The numerically longer OS observed in the older group despite shorter PFS may reflect post-progression therapy and selection bias inherent to the retrospective design; second-line therapy, predominantly paclitaxel plus ramucirumab, was administered at broadly comparable rates between the groups. Collectively, these findings suggest that nivolumab-containing regimens may provide clinically meaningful disease control in selected older patients.

Several notable differences in baseline characteristics were observed between the groups. Comorbidities were more prevalent in the ≥ 75 years group (90.0% vs. 71.2%; *P* = 0.047), as expected. Conversely, peritoneal metastasis was less frequent in patients aged ≥ 75 years (43.3% vs. 67.3%; *P* = 0.034). Given the well-established association between peritoneal metastasis and poor prognosis in gastric cancer, the lower prevalence of peritoneal metastasis in the older group may partly account for the lack of statistically significant differences in survival outcomes between the two groups [11].

Initial dose reductions of fluoropyrimidines and oxaliplatin were significantly more frequent among patients aged ≥ 75 years, and the relative dose intensity per cycle was correspondingly lower for both agents. This finding is consistent with previous studies evaluating reduced-intensity fluoropyrimidine- and oxaliplatin-based regimens in older or frail patients [12, 13]. Despite this lower dose intensity, the number of administered cycles was similar between groups, and no statistically significant differences in efficacy outcomes were observed. These findings suggest that upfront dose reduction may have facilitated treatment continuation in selected older patients, although its impact on efficacy cannot be determined from this retrospective dataset. Systematic optimization of dose-reduction strategies—ideally informed by geriatric assessment—warrants prospective evaluation in future trials dedicated to older patients, with the aim of maximizing efficacy while minimizing toxicity. Regarding safety in older patients, immunosenescence—age-related alterations in immune function—has been hypothesized to influence both the efficacy and toxicity of immune checkpoint inhibitors in this population [14, 15]. In our analysis, however, the incidences of any adverse event (AE), grade ≥ 3 AEs, any immune-related AE (irAE), and grade ≥ 3 irAEs did not differ significantly between the two groups. These findings should be interpreted cautiously, as prior studies suggest that irAE incidence does not increase uniformly with age and that age-related differences may be organ-specific rather than uniformly protective [16–18]. Notably, grade ≥ 3 AEs occurred in 53.3% of patients aged ≥ 75 years, a numerically high incidence; the absence of a statistically significant difference may reflect the limited sample size rather than true comparability.

In the exploratory multivariable analysis of the ≥ 75 years subgroup, NLR remained significantly associated with shorter PFS. NLR is a readily available marker of systemic inflammation and immune status, and its prognostic value has been reported in gastric cancer patients receiving chemotherapy and ICI-based therapy [19, 20]. Although NLR may increase with advancing age, baseline NLR was comparable between age groups in the present cohort [21]. This association was not observed for OS, possibly because OS is influenced by subsequent lines of therapy, in which ICI-containing regimens were not used. A similar association between higher NLR and shorter PFS was observed in patients aged < 75 years (HR, 1.078; 95% CI, 1.022–1.137; *P* = 0.006). Given the exploratory nature of these analyses, the small sample size, and the absence of a prespecified NLR cutoff, these findings should be interpreted with caution and validated prospectively; future biomarker reporting should adhere to REMARK guidelines [22].

Diffuse-type Lauren classification also remained significantly associated with shorter OS in the exploratory multivariable analysis. Diffuse-type gastric cancer is characterized by a cold tumor microenvironment, featuring impaired CD8 + T-cell function and elevated expression of immunosuppressive factors such as IL-10, TGF-β1, and IDO1, which may limit the efficacy of immune checkpoint blockade [23]. However, the wide confidence interval for diffuse-type histology (HR 3.451; 95% CI, 1.206–9.881) reflects the small number of events in this subgroup; the magnitude of this hazard ratio should not be overinterpreted, and this exploratory association requires prospective validation.

This study has several limitations. First, the retrospective design and relatively small sample size—particularly in the ≥ 75 years cohort (*n* = 30)—may limit statistical power and generalizability; the study was not powered to detect clinically meaningful differences between age groups. Second, chronological age alone is an insufficient measure of fitness. The absence of comprehensive geriatric assessment—including validated frailty indices, the G8 screening tool, the Charlson Comorbidity Index, sarcopenia assessment, and evaluation of activities of daily living, instrumental activities of daily living, and cognitive function—precluded thorough characterization of functional status and treatment tolerance in the older cohort. Third, comprehensive molecular profiling was not systematically performed: MSI status was assessed in only a subset of patients, and EBV status was not routinely evaluated during the study period, potentially omitting prognostic and predictive information relevant to this cohort. Fourth, because enrolled patients were treated between January 2022 and February 2025, the findings may reflect practice patterns specific to this period and may not fully represent current or evolving clinical practice. Fifth, selection bias is inherent to the retrospective design: patients who received ICI-containing combination therapy likely represent a relatively fit subset of the older population, whereas those judged unsuitable for combination therapy—even with upfront dose reduction—because of marked frailty, poor performance status, or organ dysfunction were likely underrepresented. Sixth, the use of 75 years as the threshold for defining older patients, although consistent with Japanese geriatric medicine guidelines, is an arbitrary cutoff that may not fully capture the heterogeneity of aging [8].

## Conclusions

These findings suggest the clinical feasibility of first-line nivolumab-containing chemotherapy in selected patients aged ≥ 75 years with HER2-negative unresectable advanced or recurrent gastric cancer. However, they should not be interpreted as evidence of equivalent efficacy or safety between age groups. Prospective studies incorporating comprehensive geriatric assessments are warranted to establish optimal patient selection criteria and to refine treatment strategies for this growing patient population.

## Data Availability

The datasets analyzed during the current study are not publicly available due to patient privacy and ethical restrictions. De-identified data may be made available from the corresponding author upon reasonable request and with approval from the relevant institutional review board.

## Abbreviations

AE: Adverse event
BMI: Body mass index
CI: Confidence interval
CPS: Combined positive score
CTCAE: Common Terminology Criteria for Adverse Events
DCR: Disease control rate
ECOG PS: Eastern Cooperative Oncology Group performance status
GNRI: Geriatric nutritional risk index
HER2: Human epidermal growth factor receptor 2
HR: Hazard ratio
ICI: Immune checkpoint inhibitor
irAE: Immune-related adverse event
mGPS: Modified Glasgow prognostic score
NLR: Neutrophil-to-lymphocyte ratio
ORR: Objective response rate
OS: Overall survival
PD-L1: Programmed death-ligand 1
PFS: Progression-free survival
PLR: Platelet-to-lymphocyte ratio
PNI: Prognostic nutritional index
RECIST: Response Evaluation Criteria in Solid Tumors
